# Effect of medium-chain triglycerides and whey protein isolate preloads on glycaemia in type 2 diabetes: a randomized crossover study

**DOI:** 10.1016/j.ajcnut.2024.12.022

**Published:** 2024-12-26

**Authors:** Pardeep Pabla, Joanne Mallinson, Aline Nixon, Mia Keeton, Scott Cooper, Melanie Marshall, Matthew Jacques, Sara Brown, Odd Erik Johansen, Bernard Cuenoud, Leonidas G Karagounis, Kostas Tsintzas

**Affiliations:** 1 MRC Versus Arthritis Centre for Musculoskeletal Ageing Research, School of Life Sciences, University of Nottingham, Queen’s Medical Centre, Nottingham, United Kingdom; 2 Nestlé Health Science, Vevey, Switzerland; 3 Mary MacKillop Institute for Health Research, Australian Catholic University, Melbourne, VIC, Australia; 4 Institute of Social and Preventive Medicine, University of Bern, Bern, Switzerland

**Keywords:** type 2 diabetes, glycaemia, appetite, nutritional preloads, whey protein, medium-chain triglycerides

## Abstract

**Background:**

Small nutritional preloads can reduce postprandial glucose excursions in individuals with and without metabolic syndrome or type 2 diabetes (T2D). However, most studies have focused on preloads administered before single meals and have predominantly used protein-based preloads.

**Objectives:**

To investigate the effects of sequential consumption of medium-chain triglycerides (MCT) and whey protein isolate (WPI) preloads before breakfast, lunch, and dinner on postprandial, diurnal, and 24-h glycaemia in individuals with T2D.

**Methods:**

Participants with T2D were studied over 3 randomized 24-h periods. They consumed either water before standardized breakfast, lunch, and dinner (CONTROL), 15 g MCT before breakfast and water before lunch and dinner (MCT), or 15 g MCT before breakfast and 10 g WPI before lunch and dinner (MCT + WPI). Diurnal (08:00–23:00 h) and 24 h (08:00–08:00 h) glycaemia (incremental AUC [iAUC]) and glycaemic variability (%coefficient of variation [%CV]) were evaluated by continuous glucose monitoring. Postprandial glycaemia (PPG) after breakfast and lunch was assessed by arterialized blood glucose iAUC.

**Results:**

In 21 enrolled patients (8 males/13 females, mean ± standard deviation age 55.1 ± 8.5 y, body mass index 31.7 ± 4.3 kg·m^−2^, glycated hemoglobin 59 ± 12 mmol·mol^−1^) diurnal and 24-h iAUC were similar across interventions, whereas 24-h %CV was lower in MCT (16.8 ± 0.8%, *P =* 0.033) and MCT + WPI (16.1 ± 0.9%, *P =* 0.0004) than CONTROL (18.7 ± 0.9%). PPG iAUC was ∼17% lower after breakfast in MCT and MCT + WPI compared with CONTROL, but only the MCT + WPI lowered glucose by 20% (*P =* 0.002) over the entire day (08:30–17:30 h). Gastric inhibitory polypeptide (GIP) (*P =* 0.00004), peptide YY (PYY) (*P =* 0.01), and β-hydroxybutyrate (*P =* 0.0001) were higher in MCT and MCT + WPI than CONTROL. Subjective appetite ratings were lower after breakfast and lunch in MCT + WPI (*P =* 0.001).

**Conclusions:**

Sequential consumption of MCT and WPI preloads did not affect diurnal or 24-h glycaemia but lowered PPG and 24-h glycaemic variability in individuals with T2D. These effects were associated with increased circulating β-hydroxybutyrate, PYY, and GIP, and suppression of appetite.

This trial was registered at clinicaltrials.gov as NCT04905589 (https://clinicaltrials.gov/study/NCT04905589).

## Introduction

Managing type 2 diabetes (T2D) requires considerations around reducing postprandial glycaemia (PPG) given its contribution to overall glucose burden and association with glycated hemoglobin (HbA1c), a long-term indicator of glycaemic control [1]. Because current guidelines from health organizations [2] recommend changes in dietary and physical activity patterns as important factors for management of hyperglycaemia in T2D, there is a need for innovative and feasible nutritional approaches that can reduce PPG.

There is evidence that nutritional preloads (defined as premeal consumption of specific nutrient[s]) can exert positive influence on glycaemia and appetite across a range of populations, including healthy individuals and people with obesity or T2D. Two food components that have attracted scientific interest as preloads are whey proteins, derived from dairy products, and medium-chain triglycerides (MCT), composed of fatty acids with 6–12 carbons, present in food sources such as palm kernel oil and coconut oil. Indeed, in individuals with T2D, 15 g whey protein isolate (WPI) consumed immediately or few minutes before a mixed meal resulted in lower 3-h PPG [3] and improved daily hyperglycaemia over 7 d of free living [4].

There is less knowledge regarding the impact of MCT preloads, especially in individuals with T2D, on metabolic responses. Acute ingestion of 10 g MCT 1 h before lunch was shown to lower PPG and improve satiety, leading to reduced energy intake in males with overweight [5]. Similarly, a reduction in PPG was observed in healthy individuals when 10 g MCT were coingested with a meal [6]. MCT have also been reported to lower PPG in individuals with T2D compared with long-chain triglycerides (LCT) when ingested daily over 1 mo [7]. Furthermore, short-term (5 d) partial replacement of dietary LCT with MCT improved insulin-mediated glucose metabolism in individuals with T2D, an effect that was associated with increased insulin-mediated glucose disposal and was independent of glucose-lowering therapy or the severity of fasting hyperglycaemia [8]. Several factors including increased production of ketone bodies in a dose-dependent manner [6], secretion of incretins such as peptide YY (PYY) [5], and/or potentiation of glucose-stimulated insulin secretion [9,10] may explain the beneficial influence of dietary MCT in enhancing glucose tolerance in individuals with T2D [7,8]. However, the impact of physiologically relevant amounts of MCT preloads, both independently and in combination with whey protein across different meal occasions, on subsequent postprandial and diurnal glycaemia in individuals with T2D remains unclear.

The aim of this study was to examine the effects of sequential consumption of an MCT preload at breakfast and a WPI preload at lunch and dinner on diurnal (primary outcome) and PPG (secondary outcome), as well as associated regulatory factors, in individuals with T2D. Other secondary aims included assessing the impact of these preloads on markers of 24-h glycaemia and appetite.

## Methods

### Participants

Male and female participants of 25–65 y of age, with a BMI of ≤40 kg^.^m^−^^2^, and diagnosis of T2D documented by either HbA1c 48–86 mmol·mol^−1^ or on oral glucose-lowering medication (either monotherapy or stable oral combination therapies) were recruited. Individuals were excluded if they had fasting blood glucose >11 mmol·L^−1^ at screening; ongoing or recent (<3 mo) treatment with injectable glucose-lowering medications [for example, insulin and glucagon-like peptide 1 (GLP1)]; weight loss interventions; history of bariatric surgery or eating disorders; ongoing treatment with anorectic medications, steroids, medications known to affect gastric motility, or any condition affecting gastro-intestinal integrity; history of smoking, excessive alcohol consumption (>28 units·wk^−1^) and gastrointestinal, liver, cardiovascular, metabolic/or endocrine disorders other than T2D.

After written informed consent (the study was approved by the East Midlands/Nottingham1 Research Ethics Committee and Health Research Authority, reference No 21/EM/0005), participants undertook a medical screening. It comprised of medical history, health questionnaires, 12-lead electrocardiogram, blood pressure, weight and height measurements, and blood and urine samples for routine blood chemistry, hematology, and urinalysis. Once eligibility was confirmed, habitual physical activity (3-d wrist worn triaxial accelerometers; GENEActiv, Activinsights) and dietary (3-d weighted food diaries) patterns were assessed.

### Experimental design

We utilized a single-center, randomized, open-label, 3-arm, crossover study (clinicaltrials.gov identifier NCT04905589). Participants attended the Human Physiology Unit at the University of Nottingham on 3 separate occasions, ≥1 wk apart, and underwent 3 interventions in a randomized (1:1:1 ratio) crossover design. Each participant was assigned 1 of 3 randomized treatment arms, each following a specific sequence. Participants in arm 1 followed the sequence ABC, those in arm 2 followed BCA, and participants in arm 3 followed CAB, where A represented CONTROL, B represented MCT, and C represented MCT + WPI. On each visit, participants consumed either: *1*) 15 g of MCT in liquid form (75 mL) 30 min before breakfast and 10 g of whey protein (dissolved in 200 mL of water) 30 min before lunch and 30 min before dinner (MCT + WPI treatment); or *2*) iso-voluminous water for each corresponding preload 30 min before breakfast (75 mL), lunch (200 mL), and dinner (200 mL) (CONTROL); or *3*) 15 g of MCT 30 min before breakfast and 200 mL of water before lunch and dinner (MCT). MCT was consumed in the form of 75 mL of BetaQuik (currently branded as K·Quik) (Vitaflo International), containing 54% caprylic acid, 43% capric acid, and 2.5% lauric acid. Whey protein was consumed in the form of 12.5 g of WheyBasics (Pure Encapsulations) [providing 10 g of WPI, of which 2.2 g in the form of branched-chain amino acids (BCAA)] dissolved in 200 mL of water. Breakfast, lunch, and dinner were standardized for each participant across study days according to their isoenergetic daily energy requirements, and their cumulative composition across the day provided 26.9 ± 0.6% of total daily energy from fat, 52.7 ± 0.4% from carbohydrate (CHO), and 18.1 ± 0.1% from protein. The distribution of energy across the 3 meals was 20% from breakfast (consumed at 09:00), 35% from lunch (consumed at 13:30), and 35% from dinner (consumed at 18:00). A standardized snack (10% of total daily energy intake) was provided at 21:00 to meet the daily energy requirements for each participant. Details on energy intake and macronutrient composition of test meals are included in Table 1.

**TABLE 1:** Mean energy intake and macronutrient composition of test meals for all participants.

	Breakfast	Lunch	Dinner	Snack	Total
Energy (kJ)	2100 ± 312	3675 ± 545	3675 ± 545	1050 ± 156	10,501 ± 1558
CHO (g)	78 ± 12	111 ± 16	129 ± 19	13 ± 2	331 ± 49
CHO (%E)	62.2 ± 1.1	50.1 ± 0.3	58.8 ± 0.1	21.0 ± 0.7	52.7 ± 0.4
Fat (g)	9 ± 1	24 ± 4	23 ± 3	18 ± 3	74 ± 11
Fat (%E)	17.1 ± 0.8	26.2 ± 0.4	23.4 ± 0.1	63.2 ± 0.7	26.9 ± 0.6
Protein (g)	18 ± 3	46 ± 7	40 ± 6	9 ± 1	114 ± 17
Protein (%E)	14.6 ± 0.6	21.9 ± 0.1	18.3 ± 0.1	14.6 ± 0.1	18.1 ± 0.1

Values are means ± SD; *n =* 21.

Abbreviations: %E, percent contribution to daily energy intake; CHO, carbohydrate.

### Experimental protocol

**FIGURE 1:**
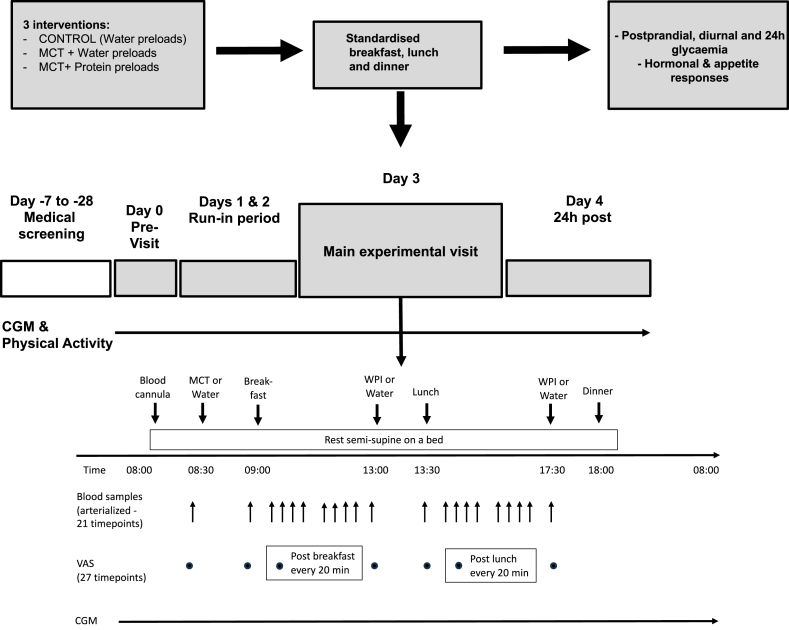
Schematic diagram of experimental protocol. CGM, continuous glucose monitoring; MCT, medium-chain triglycerides; VAS, visual analogue scale; WPI, whey protein isolate.

The schematic diagram of the experimental protocol is included in Figure 1. All participants attended the laboratory on 6 separate occasions: 3 main intervention visits (visits 2, 4, and 6), and 3 short visits at 3–5 d before each main intervention visit (previsits 1, 3, and 5). The washout period after each main intervention visit was between 1 and 8 wk. Participants underwent a 2-d run-in period before each main treatment visit. During previsits 1, 3, and 5, participants were fitted with a subcutaneous continuous glucose monitoring (CGM) device (FreeStyle Libre, Abbott Diabetes Care), which measured interstitial glucose concentrations every 15 min throughout the run-in period and the intervention day visit. During the 2-d run-in period, participants were asked to refrain from strenuous exercise and alcohol and consume standardized meals (menus were provided to them by the investigators) that were isoenergetic relative to individual daily energy requirements calculated from estimated resting metabolic rates using Henry equations [11] and habitual levels of physical activity. During previsit 1 only, dual energy X-ray absorptiometry scans (Lunar Prodigy, GE Healthcare) were performed to assess body composition.

On each intervention visit (visits 2, 4, and 6), participants reported to the laboratory at 08:00, after an overnight fast (11 h). A cannula was then inserted retrograde into a superficial dorsal vein of the dominant hand, which was kept in a warming unit (55°C) to arterialize the venous drainage [12] and was kept patent via a saline drip. On all 3 visits, participants consumed a standardized breakfast at 09:00, lunch at 13:30, and dinner at 18:00 and preloads (MCT, WPI, or water) 30 min before each meal (at 08:30, 13:00, and 17:30, respectively). Blood samples were obtained before consumption of the first preload (*t* = −30 min), immediately before breakfast (*t* = 0 min), 15, 30, 45, 60, 90, 120, 150, 180, and 240 min after breakfast, immediately before lunch (*t* = 270 min), and 15, 30, 45, 60, 90, 120, 150, 180, and 240 min after lunch. A 100-point visual analogue scale (VAS) on subjective appetite and satiety ratings [13] was administered at *t* = −30 min, *t* = 0 min, every 20 min after breakfast for 4 h, at = 270 min, and every 20 min for 4 h after lunch.

### Blood analysis

Whole-blood glucose concentrations were determined using a Yellow Springs Instrument analyzer (2300 STAT PLUS, Yellow Springs Instruments). Serum was obtained from 1 aliquot of blood and analyzed for insulin concentrations by enzyme-linked immunoassay (EZHI-14K; Merck Millipore), β-hydroxybutyrate by a colorimetric enzymatic assay (ab272541; Abcam), and proinsulin by enzyme-linked immunoassay (DPINS0; Biotechne/R&D Systems). Another aliquot of blood was collected into a lithium heparin tube containing 30 μL EGTA-glutathione and centrifuged immediately (15 min, 3000 g at 4°C) to obtain plasma for the determination of total PYY by enzyme-linked immunoassay (EZHPYYT66K; Merck Millipore), c-peptide by enzyme-linked immunoassay (DICP00; Biotechne/R&D Systems), and amino acid concentrations using hydrophilic interaction liquid chromatography coupled to high-resolution mass spectrometry as previously described [14]. Two separate aliquots of blood were transferred to an EDTA tube containing a protease inhibitor (100 μL of aprotinin) to obtain plasma for the determination of glucagon by enzyme-linked immunoassay (DGCG0; Biotechne/R&D Systems), and an EDTA tube containing dipeptidyl peptidase-4 inhibitor (10 μL·mL^−1^ plasma) for the determination of total gastric inhibitory polypeptide (GIP) by enzyme-linked immunoassay (EZHGIP-54K; Merck Millipore), and active GLP1 by enzyme-linked immunoassay (EGLP-35K; Merck Millipore).

### Outcomes

The primary outcome was diurnal glucose incremental (from baseline) AUC (iAUC) (08:00–23:00) assessed by CGM, whereas the secondary outcome was defined as PPG (mean and iAUC responses) after breakfast and lunch assessed by arterialized blood glucose responses. Other secondary outcomes were changes in circulatory (mean and iAUC responses) insulin, c-peptide, proinsulin, glucagon, GIP, GLP1, PYY, and amino acids; indices of 24-h glycaemia (iAUC and glycaemic variability); and appetite scores. The statistical power analysis indicated that a total of 17 participants (completers) were required to detect a 10% reduction in diurnal glucose iAUC between MCT + WPI and CONTROL with a power of 90% at 5% significance level.

### Data handling and statistical analysis

Food diaries were kept by participants for 3 full days during the baseline period and for 2 d during the run-in to each treatment visit. Dietary analysis was performed using Nutritics (version 5.096; Nutritics Ltd). Daily energy intake (E) and CHO, fat, and protein content (in g and %E) of all meals and snacks were calculated.

CGMs were recorded for 3 full days during the run-in period and each treatment visit. Recording started ≥72 h (day 0) before participants reported to the laboratory at 08:00 on the day of each main visit (day 3) and terminated at 08:00 on the day after the visit (day 4). Only data for the last 48 h of the run-in period (to avoid bias due to insertion of the sensor and allow sufficient stabilization of the CGM system) and the 24 h of the main visit day were analyzed. CGM data were shifted back to the nearest 15-min time point, and days with ≥20% missing data were excluded. The coefficient of variation (CV) for interstitial glucose concentrations, an index of glucose variability that allows comparisons between individuals with different mean glycaemic values, was calculated as % using the formula: (SD/mean glucose) × 100.

Physical activity data were recorded at 15-s epochs using the GENEActiv software V3.2 and analyzed using an open access macro [15], which assesses physical activity patterns (sedentary, light, moderate, and vigorous activities in min) using cut-points established by Esliger et al. [16].

Subjective appetite ratings obtained from VAS were used to calculate the composite appetite score (CAS) as follows: CAS = (hunger + desire to eat + prospective food consumption + [100 – fullness] + [100 – satisfaction]) / 5 [17].

β-Cell function was estimated for the postprandial periods after breakfast and lunch using the formula: (totalAUC for insulin/totalAUC for glucose) × Matsuda insulin sensitivity index [18].

Statistical analysis was performed in GraphPad Prism (version 9.2.0; GraphPad). Descriptive data are presented as mean ± SD. Experimental data are presented as mean ± SEM and were tested for normality using the Shapiro–Wilk test. Data from all 21 participants were analyzed (with intention to treat) using a general linear mixed effects model approach without any ad hoc imputations to account for the missing values. A mixed-design 1-way analysis of variance (ANOVA) was used when the intervention was the only independent variable, and where time was also a variable; mixed-design 2-way ANOVA (main treatment × sampling time). All post hoc multiple comparisons after ANOVA that showed significant effects were undertaken using Bonferroni tests. iAUC and totalAUC were calculated using the trapezoidal method. Statistical significance was defined as *P* < 0.05.

## Results

**FIGURE 2:**
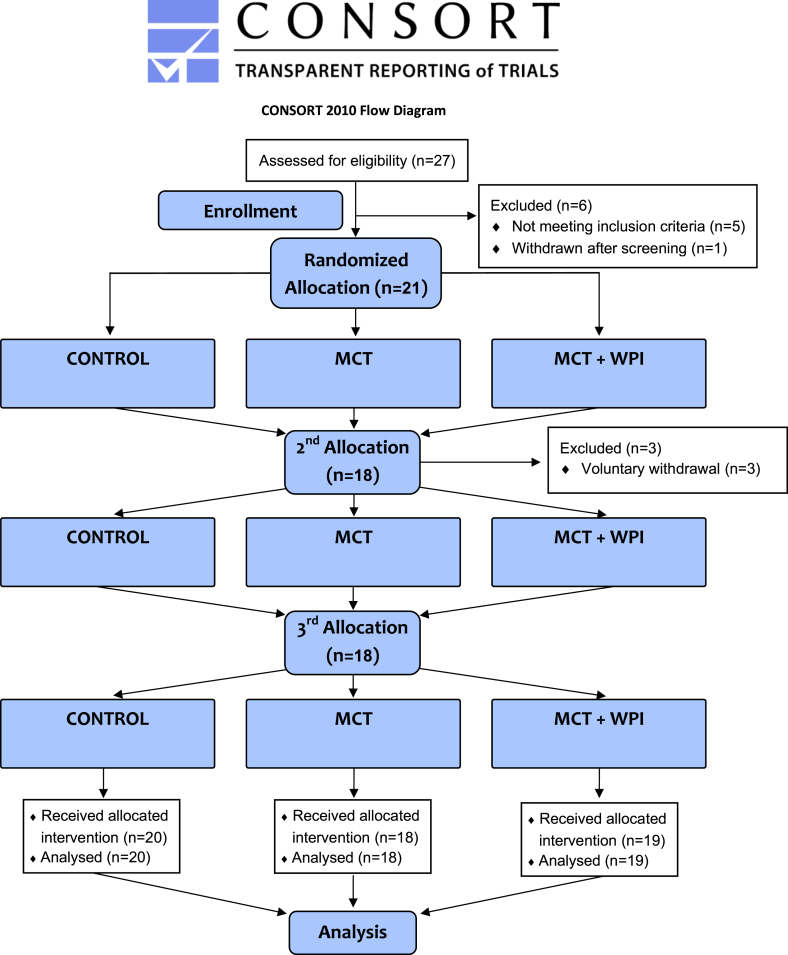
Study CONSORT flow diagram. MCT, medium -chain triglycerides; WPI, whey protein isolate.

Twenty seven participants were assessed for eligibility (Figure 2) and 21 were randomly divided and treated between 12 April, 2021, and 31 July, 2022. Baseline demographics of the 21 participants are provided in Table 2. Of the 21 randomly divided participants, 18 completed all 3 interventions and 3 participants completed 1 intervention (Figure 2).

**TABLE 2:** Participant characteristics (*n* = 21).

	Mean ± SD	Range
Age (y)	55.1 ± 8.5	29–65
Sex (*n*: Females/Males)	8/13	—
Height (m)	1.71 ± 0.10	1.56–1.89
Weight (kg)	93.2 ± 16.1	61.5–128.6
BMI (kg·m^−2^)	31.7 ± 4.3	24.5–40.0
Fat mass (kg)	35.2 ± 11.0	18.6–52.8
Lean body mass (kg)	54.6 ± 9.7	36.8–72.7
Body fat (%)	37.5 ± 7.9	25–47
Duration (time since first diagnosis) of type 2 diabetes (y)	7.5 ± 5.2	0.5–17
Fasting glucose (mmol·L^−1^)	6.5 ± 1.5	4.4–10.9
HbA1c (mmol·mol^−1^)	59 ± 12	45–93
Diabetes medication (*n*)	Metformin (12)	—
	Metformin + SU (2)	
	Metformin + DPP4i (1)	—
	Metformin + SU + DPP4i (2)	—
	Metformin + DPP4i + TZD (1)	—
	Metformin + SGLT2i (1)	—
	Metformin + SGLT2i + DPP4i (1)	—
	Diet + Lifestyle (1)	—

Abbreviations: DPP4i, dipeptidyl peptidase-4 inhibitor; HbA1c, glycated hemoglobin A1c; SGLT2i, sodium–glucose cotransporter 2 inhibitor; SU, sulfonylurea; TZD, thiazolidinediones.

### Run-in period

During the 2-d run-in period to each intervention visit, participants consumed diets that were similar between interventions and had the same macronutrient composition (Table 3). The meals consumed at 21:00 the evening before each intervention visit had the same energy and macronutrient composition (Table 3). Physical activity patterns were similar between interventions for sedentary time, light, moderate, and vigorous activities (Table 3). Interstitial glucose profiles (mean 24 h values of both days) and glycaemic variability (mean %CV) were also similar between the 2 run-in days and across all interventions (Table 3).

**TABLE 3:** Dietary, physical activity, and interstitial glucose profiles during the 2-d run-in period to each intervention visit.

	CONTROL (*n* = 20)	MCT (*n* = 18)	MCT + WPI (*n* = 19)
Daily average of dietary intake during the 2-d run-in period			
Energy (kJ)	10,172 ± 535	10,096 ± 579	10,161 ± 512
CHO (%E)	47 ± 1	47 ± 1	46 ± 1
Fat (%E)	31 ± 1	31 ± 1	32 ± 1
Protein (%E)	22 ± 1	22 ± 1	23 ± 1
Composition of meals consumed the evening before each intervention			
Energy (kJ)	4242 ± 279	4426 ± 272	4447 ± 565
CHO (%E)	41 ± 2	40 ± 2	39 ± 2
Fat (%E)	34 ± 2	35 ± 2	36 ± 3
Protein (%E)	25 ± 2	25 ± 2	25 ± 3
Daily average of physical activity patterns during the 2-d run-in period			
Sedentary time (min)	724 ± 21	695 ± 20	697 ± 25
Light activities (min)	190 ± 12	185 ± 15	191 ± 14
Moderate activities (min)	78 ± 9	78 ± 9	82 ± 9
Vigorous activities (min)	2 ± 1	2 ± 1	2 ± 1
Interstitial glucose profiles during the 2-d run-in period			
Mean 24 h (mmol^.^L^−1^)	8.41 ± 0.38	8.63 ± 0.40	8.53 ± 0.36
Mean 24-h %CV	17.8 ± 1.2	17.9 ± 1.0	18.7 ± 0.9

Values are means ± SEM.

Abbreviations: %CV, percent coefficient of variation; %E, percent contribution to daily energy intake; CHO, carbohydrate; MCT, medium-chain triglyceride; WPI, whey protein isolate.

### Main experimental interventions—continuous glucose monitoring

**FIGURE 3:**
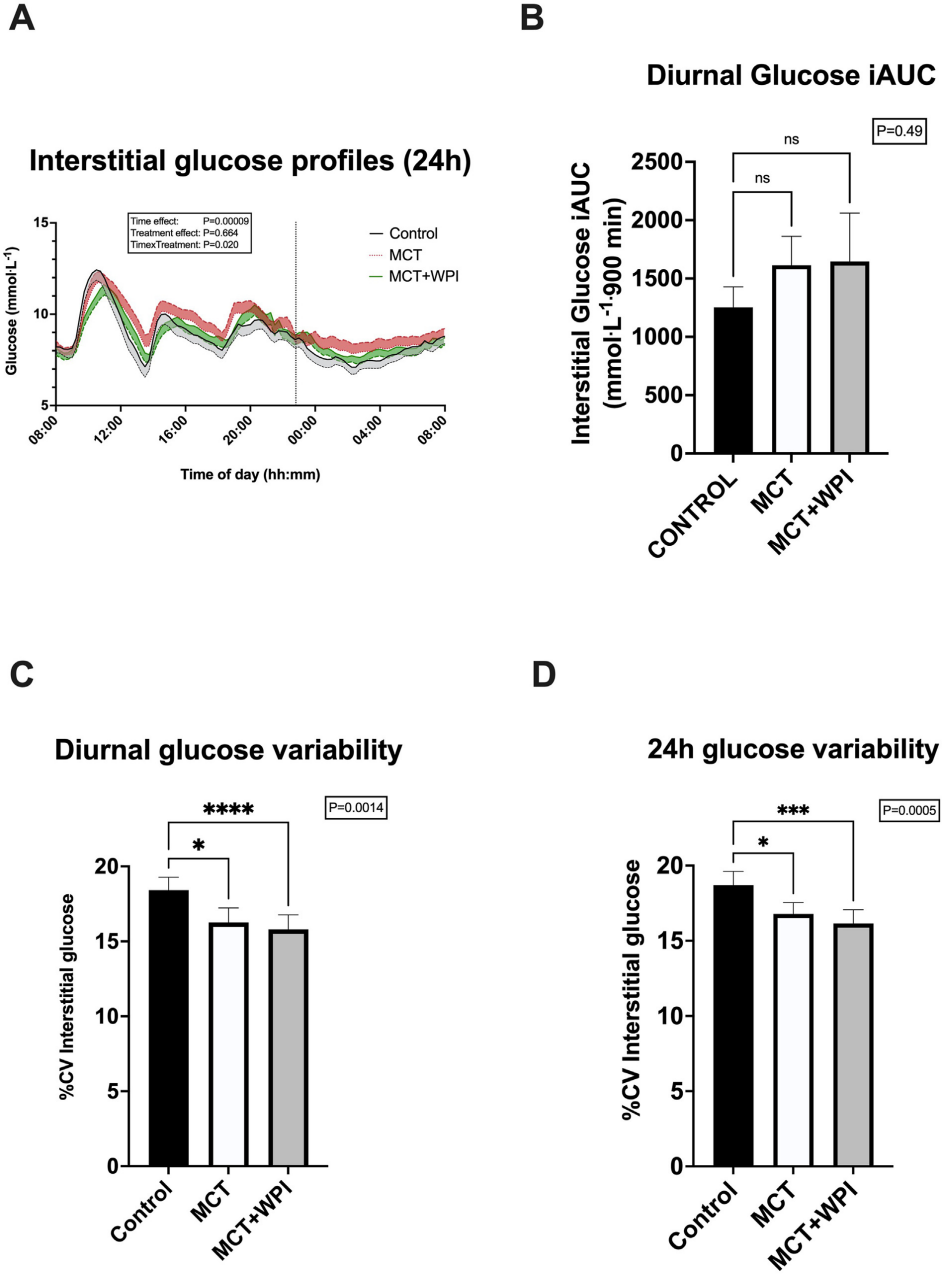
Diurnal and 24-h interstitial glucose responses. Effects of water consumption 30 min before breakfast, lunch, and dinner (CONTROL, *n =* 20), MCT consumption before breakfast and water before lunch and dinner (MCT, *n =* 18), or MCT consumption before breakfast and WPI before lunch and dinner (MCT + WPI, *n =* 19), on 24-h interstitial glucose profiles (A), diurnal (08:00–23:00) glucose iAUC (B), percent CV of diurnal (08:00–23:00) glucose concentrations (C) and percent CV of 24-h glucose concentrations (D). Values are means ± SEM. Main effects of 1- and 2-way ANOVA are displayed in text boxes. Post hoc comparisons: ∗*P <* 0.05; ∗∗∗*P <* 0.001; ∗∗∗∗*P <* 0.0001 from CONTROL. %CV, percent coefficient of variation; ANOVA, analysis of variance; iAUC, incremental AUC; MCT, medium-chain triglycerides; WPI, whey protein isolate.

Interstitial glucose profiles for the main interventions are displayed in Figure 3A. Analysis of absolute glucose concentrations revealed a significant treatment × sampling time interaction (*P* = 0.020), which was driven by attenuated glucose responses in the early postprandial period after breakfast in MCT and MCT + WPI but higher responses later in the day when compared with CONTROL (Figure 3A). As a result, there were no significant differences in diurnal (08:00–23:00) (Figure 3B), 24-h (08:00–08:00) glucose iAUC or mean diurnal (CONTROL 9.28 ± 0.45 mmol^.^L^−1^; MCT 9.49 ± 0.48 mmol^.^L^−1^; MCT + WPI 9.41 ± 0.41 mmol^.^L^−1^), and 24-h (CONTROL 8.74 ± 0.41 mmol^.^L^−1^; MCT 9.00 ± 0.46 mmol^.^L^−1^; MCT + WPI 8.94 ± 0.37 mmol^.^L^−1^) glucose concentrations between interventions. However, glycaemic variability (%CV) was lower in both MCT (24 h: 16.8 ± 0.8%, *P =* 0.033; diurnal: 16.3 ± 1.0%, *P =* 0.024) and MCT + WPI (24 h: 16.1 ± 0.9%, *P =* 0.0004; diurnal: 15.8 ± 1.0%, *P =* 0.00008) than CONTROL (24 h: 18.7 ± 0.9%; diurnal: 18.4 ± 0.9%) (Figure 3C and D).

### Postprandial glucose

**FIGURE 4:**
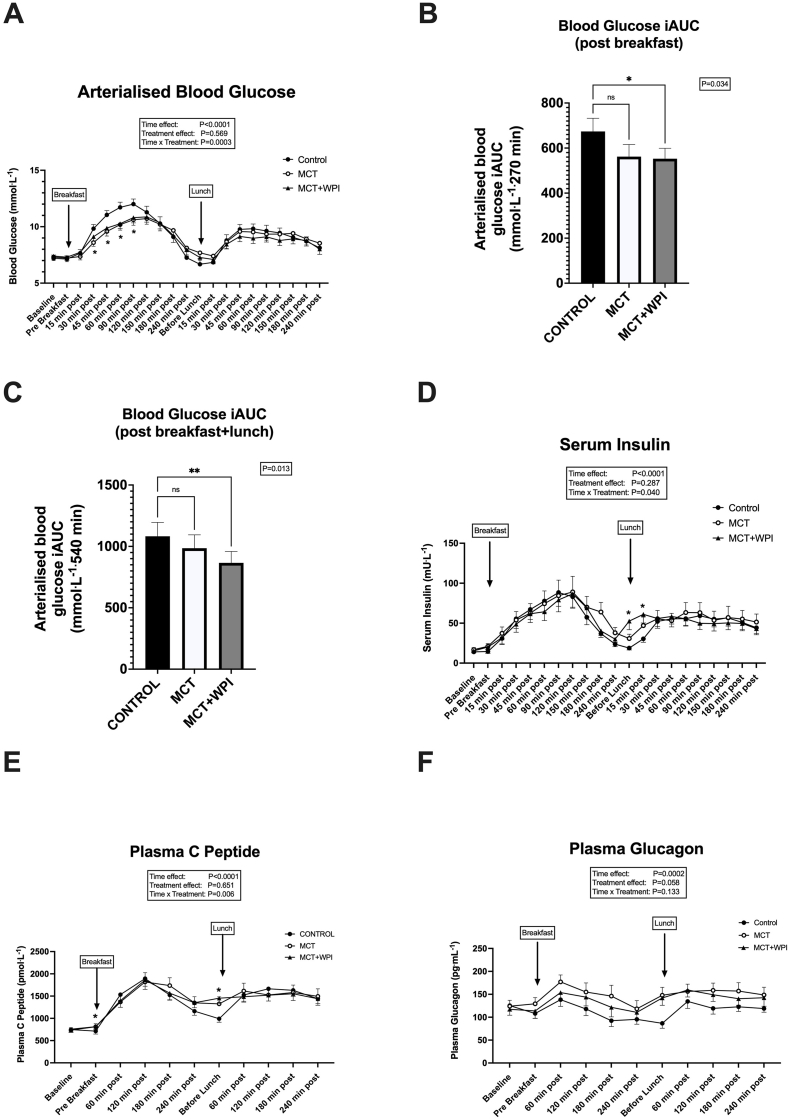
Postprandial blood glucose responses. Effects of water consumption 30 min before breakfast, lunch, and dinner (CONTROL, *n =* 20), MCT consumption before breakfast and water before lunch and dinner (MCT, *n =* 18), or MCT consumption before breakfast and WPI before lunch and dinner (MCT + WPI, *n =* 19), on diurnal (08:30–17:30) postprandial arterialized glucose concentrations (A), postprandial breakfast (08:30–13:00) arterialized glucose iAUC (B), postprandial diurnal (08:30–17:30) arterialized glucose iAUC (C), postprandial arterialized serum insulin concentrations (D), postprandial arterialized plasma c-peptide concentrations (E), and postprandial arterialized plasma glucagon concentrations (F). Values are means ± SEM. Main effects of 2- and 1-way ANOVA are displayed in text boxes. Post hoc comparisons: ∗*P <* 0.05; ∗∗*P <* 0.01; ∗∗∗*P <* 0.001 from CONTROL. ANOVA, analysis of variance; iAUC, incremental AUC; MCT, medium-chain triglycerides; WPI, whey protein isolate.

After consumption of breakfast and lunch, arterialized glucose concentrations (Figure 4A) increased sharply in all trials (main effect of sampling time, *P <* 0.0001). However, there was a significant treatment × sampling time interaction (*P =* 0.0003), which was driven by attenuated glucose responses after breakfast in both MCT and MCT + WPI when compared with CONTROL but only in MCT + WPI after lunch (Figure 4A). Despite a similar reduction of ∼17% in glucose iAUC after breakfast in both MCT and MCT + WPI (*P =* 0.05 and *P =* 0.01, respectively) (Figure 4B), when considered over the entire breakfast and lunch postprandial period (08:30–17:30) only the MCT + WPI showed lower (20%, *P =* 0.002) glucose iAUC compared with CONTROL (Figure 4C).

### Postprandial hormonal responses

A significant intervention × sampling time interaction (*P =* 0.04) was observed for serum insulin levels (Figure 4D), which increased after consumption of breakfast and lunch and were similar between CONTROL and MCT, but the early postprandial response after the lunch preload was higher in MCT + WPI than CONTROL (*P =* 0.003). However, this effect was transient and did not affect the insulin iAUC when considered over the entire postprandial lunch period (data not shown). In accordance, the estimated β-cell function index was similar between interventions both after breakfast (CONTROL 15.2 ± 1.6; MCT 16.2 ± 1.9; MCT + WPI 14.2 ± 1.5) and lunch (CONTROL 15.2 ± 2.0; MCT 13.2 ± 2.3; MCT + WPI 13.4 ± 1.9).

Plasma c-peptide concentrations increased with all interventions after consumption of breakfast and lunch (main effect of sampling time, *P <* 0.0001) (Figure 4E). There was a significant intervention × sampling time interaction (*P =* 0.006), which was driven by higher values immediately before breakfast and lunch in the MCT and MCT + WPI than CONTROL (*P =* 0.018 and *P <* 0.0001, respectively). However, these effects were transient and did not affect the c-peptide iAUC when considered over the entire day (08:30–17:30) or over the individual breakfast and lunch postprandial periods (data not shown).

Plasma glucagon concentrations increased in all trials after consumption of breakfast and lunch (main effect of sampling time, *P =* 0.0002) and tended to be higher in the MCT and MCT + WPI than CONTROL (intervention effect, *P =* 0.058) (Figure 4F). As a result, plasma glucagon iAUC was higher (*P =* 0.01) in both MCT (17,864 ± 4598 pg^.^mL^−1.^540 min) and MCT + WPI (14,851 ± 3304 pg^.^mL^−1.^540 min) than CONTROL (4945 ± 1445 pg^.^mL^−1.^540 min) when considered over the entire day (08:30–17:30).

**FIGURE 5:**
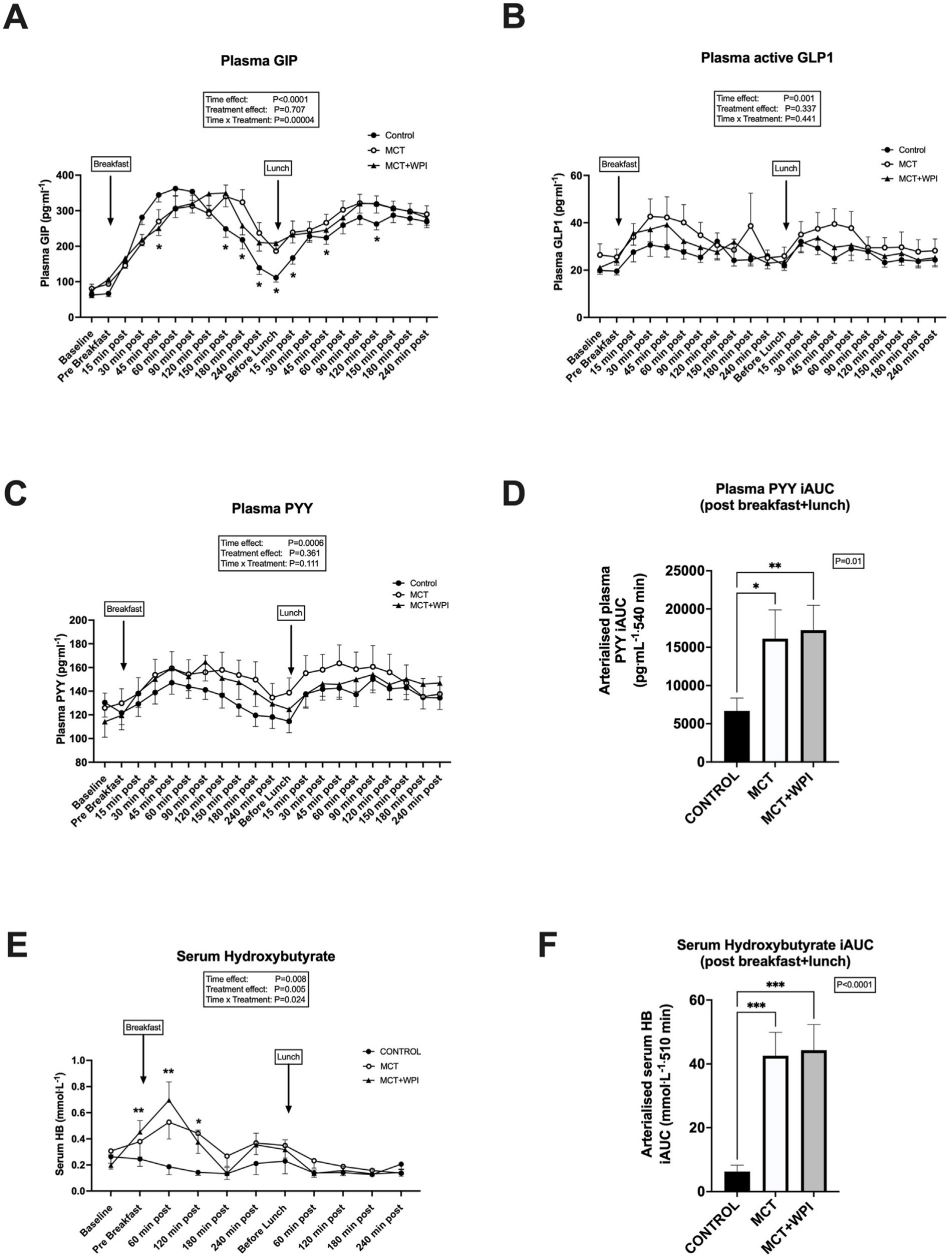
Postprandial serum hydroxybutyrate and hormonal responses. Effects of water consumption 30 min before breakfast, lunch, and dinner (CONTROL, *n =* 20), MCT consumption before breakfast and water before lunch and dinner (MCT, *n =* 18), or MCT consumption before breakfast and WPI before lunch and dinner (MCT+WPI, *n =* 19) on diurnal (08:30–17:30) postprandial arterialized plasma GIP concentrations (A), plasma active GLP1 concentrations (B), plasma PYY concentrations (C), diurnal plasma PYY iAUC (D), serum β-hydroxybutyrate concentrations (E), and serum β-hydroxybutyrate iAUC (F). Values are means ± SEM. Main effects of 2- and 1-way ANOVA are displayed in text boxes. Post hoc comparisons: ∗*P <* 0.05; ∗∗*P <* 0.01; ∗∗∗*P <* 0.001 from CONTROL. ANOVA, analysis of variance; GIP, gastric inhibitory polypeptide; GLP-1, glucagon-like peptide 1; iAUC, incremental AUC; MCT, medium-chain triglycerides; PYY, peptide YY; WPI, whey protein isolate.

Plasma GIP concentrations were higher (interaction effect *P =* 0.00004) in both MCT and MCT + WPI than CONTROL during the late postprandial period after breakfast and the early period after lunch (Figure 5A). However, there was no difference between interventions in GIP iAUC when considered over the entire day (08:30–17:30).

Plasma GLP1 concentrations increased after consumption of breakfast and lunch (main effect of sampling time, *P =* 0.001). However, there was no difference in GLP1 responses between interventions (Figure 5B). When only the 18 completers were considered, there was a tendency for higher responses in the MCT and MCT + WPI compared with CONTROL (treatment effect *P =* 0.07).

Plasma PYY concentrations increased after consumption of breakfast and lunch (main effect of sampling time, *P =* 0.0006) (Figure 5C). Plasma PYY iAUC for the combined postprandial period after breakfast and lunch was higher in both MCT (*P =* 0.017) and MCT + WPI (*P =* 0.009) than CONTROL (Figure 5D).

### Postprandial β-hydroxybutyrate

Serum β-hydroxybutyrate concentrations increased after consumption of breakfast in both MCT and MCT + WPI interventions but remained unchanged in CONTROL (interaction effect *P =* 0.024; Figure 5E). Serum β-hydroxybutyrate iAUC was higher in both MCT and MCT + WPI than CONTROL (*P =* 0.0004) when considered over the entire day (08:30–17:30) (Figure 5F).

### Postprandial amino acids

**FIGURE 6:**
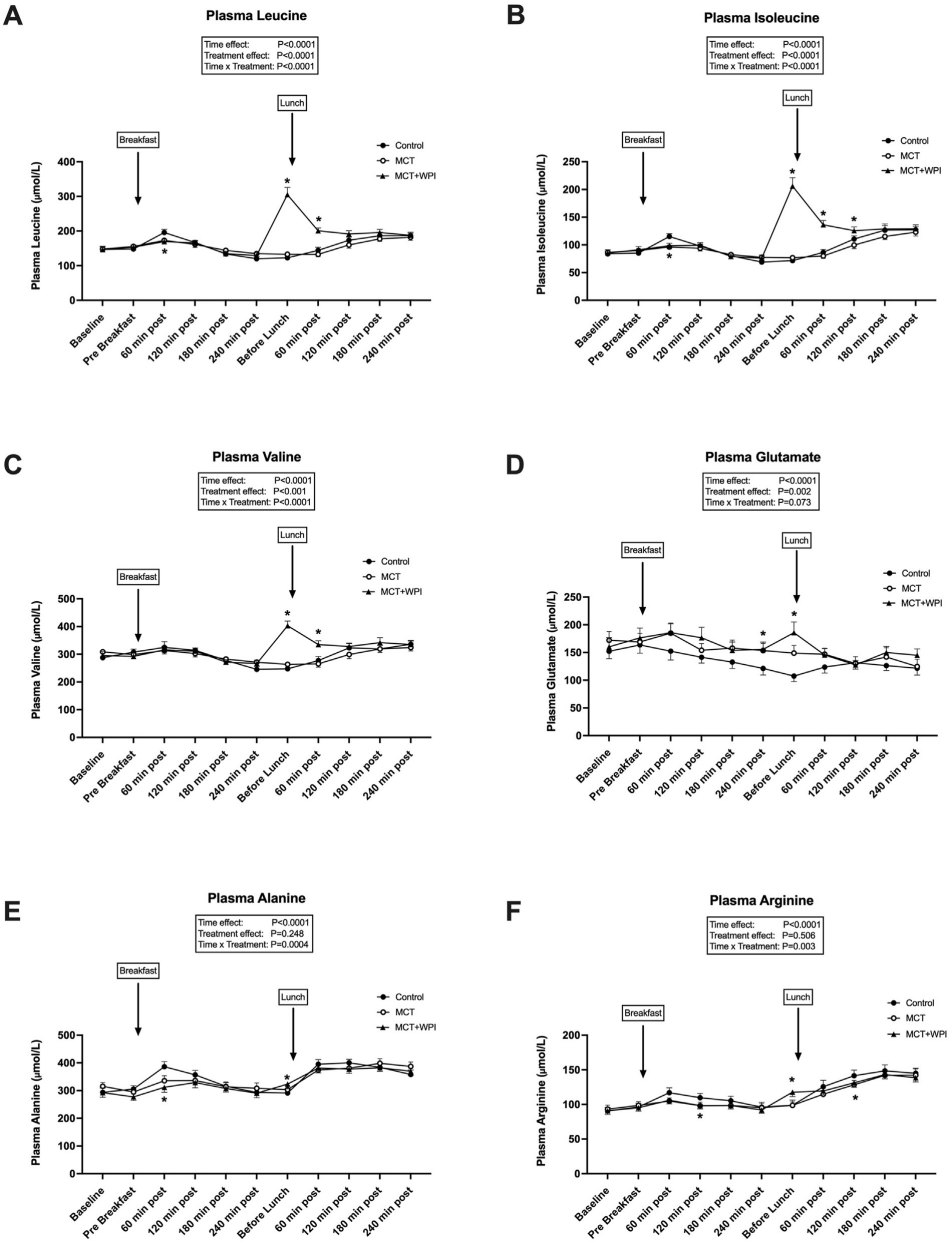
Postprandial arterialized plasma amino acids responses. Effects of water consumption 30 min before breakfast, lunch, and dinner (CONTROL, *n =* 20), MCT consumption before breakfast and water before lunch and dinner (MCT, *n =* 18), or MCT consumption before breakfast and WPI before lunch and dinner (MCT + WPI, *n =* 19), on diurnal (08:30–17:30) arterialized plasma leucine, isoleucine, valine, glutamate, alanine, and arginine concentrations (A–F). Values are means ± SEM. Main effects of 2-way ANOVA are displayed in the text box. Post hoc comparisons: ∗*P <* 0.05 from CONTROL. ANOVA, analysis of variance; MCT, medium-chain triglycerides; WPI, whey protein isolate.

Plasma BCAA concentrations increased after ingestion of WPI and remained elevated for the first hour after ingestion of lunch in the MCT + WPI trial only (interaction effects, *P <* 0.0001) (Figure 6A–C). Similar responses were observed for glutamate, alanine, and arginine concentrations (Figure 6D–F).

In a per protocol analysis (based on 18 completers) of all blood responses, the results were similar to the statistical outcomes of the intention to treat analysis (data not shown).

### Appetite

**FIGURE 7:**
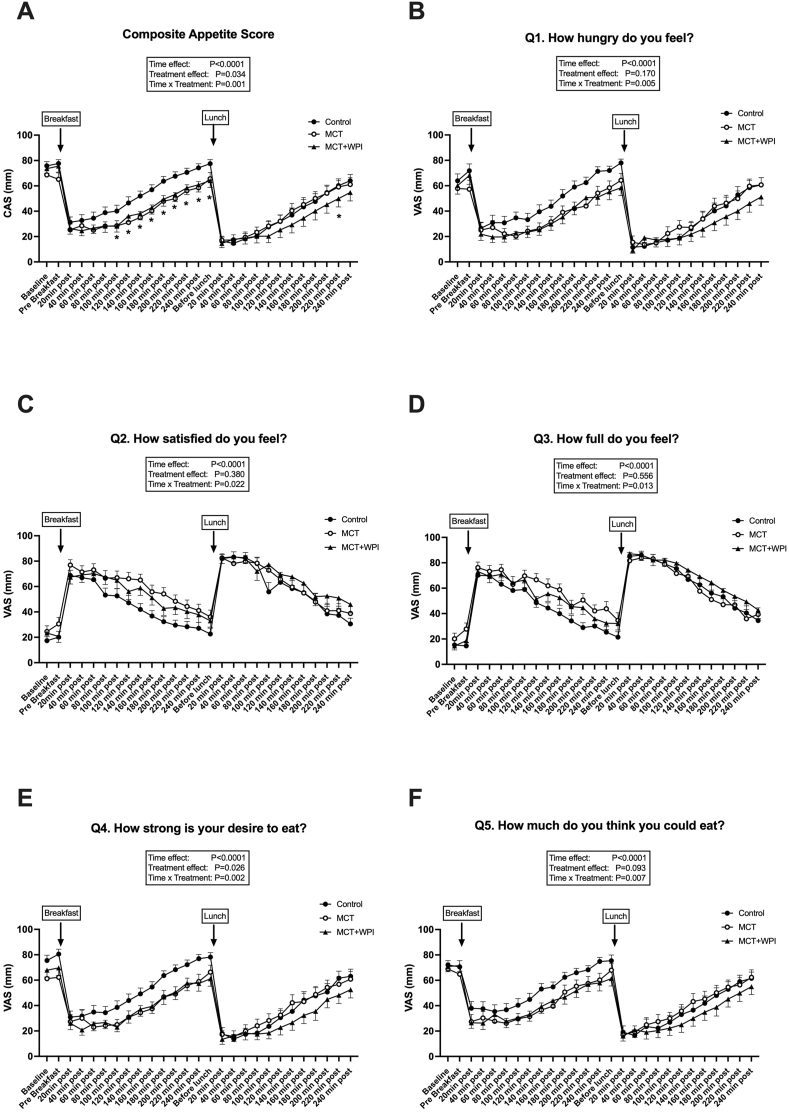
Subjective markers of appetite. Effects of water consumption 30 min before breakfast, lunch, and dinner (CONTROL, *n =* 20), MCT consumption before breakfast and water before lunch and dinner (MCT, *n =* 18), or MCT consumption before breakfast and WPI before lunch and dinner (MCT + WPI, *n =* 19), on diurnal (08:30–17:30) postprandial composite appetite scores (A) and subjective appetite responses (B–F). Values are means ± SEM. Main effects of 2-way ANOVA are displayed in the text box. Post hoc comparisons: ∗*P <* 0.05 from CONTROL. ANOVA, analysis of variance; CAS, composite appetite score; MCT, medium-chain triglycerides; VAS, visual analogue scale; WPI, whey protein isolate.

The CASs were lower after breakfast in MCT and MCT + WPI compared with CONTROL, whereas after lunch only MCT + WPI had lower values (treatment and interaction effects, *P =* 0.034 and *P =* 0.001, respectively) (Figure 7A). Individual CAS components showed decreased hunger and increased fullness (Figure 7B–F).

### Adverse events

Interventions and experimental approaches were well tolerated, and no serious adverse events were reported.

## Discussion

Sequential oral ingestion of MCT and WPI meal preloads over the course of 1 d did not affect diurnal or 24-h glycaemia in individuals with T2D but demonstrated a reduction in PPG and 24-h glycaemic variability. These effects were associated with increased circulating insulin (transiently after lunch), ketones (β-hydroxybutyrate), PYY, GIP, and glucagon, and suppression of appetite.

Variations in absolute interstitial glucose concentrations were characterized by the MCT-induced transient attenuation in glucose responses during the early postprandial period after breakfast, followed by elevated responses later in the day compared with control conditions. This response pattern accounted for similar diurnal (the primary outcome of this study) and 24-h iAUC responses observed across interventions. Although there was no overall effect on diurnal and 24-h glycaemia, this study revealed that consuming MCT and whey protein preloads reduces 24-h glycaemic variability. The clinical importance of glycaemic variability extends to overall glycaemic control [19,20] and microvascular complications in patients with T2D [20]. Interestingly, in this study 24-h glycaemic variability showed an improvement in the MCT intervention to an extent comparable with the MCT + WPI, indicating that MCT consumption at breakfast might be the primary driver for this potentially beneficial effect from preloads.

Reducing PPG is important in the management of T2D because of its integral relationship with chronic glycaemic control [1,21]. In this study, a similar reduction in glucose iAUC was observed after breakfast in MCT and MCT + WPI, whereas a prolonged attenuation of PPG throughout the day (08:30–17:30) was evident only in MCT + WPI when compared with CONTROL.

The mechanisms underpinning the glucose-lowering effect of MCT preloads are not precisely defined. MCT may reduce PPG by enhancing insulin secretion, potentially through increased production of ketone bodies [22] and/or secretion of incretins such as PYY, but not GLP1 [5]. MCTs are primarily oxidized in the liver and their consumption results in increased production and prolonged release in the blood (for ≤4–5 h) of ketone bodies in a dose-dependent manner [6]. In this study, consumption of 15 g of MCTs before breakfast resulted in ∼7-fold increase in circulating β-hydroxybutyrate levels (the most abundant ketone body in humans) that persisted during the postprandial lunch period.

In rodent islet models, caprylic acid (the primary component of MCT) has been shown to potentiate glucose-stimulated insulin secretion via a calcium dependent pathway [10], whereas 6 wk of MCT supplementation (30 g per 2000 kcal of daily energy intake) as a substitution for equivalent amount of fat intake demonstrated inconsistent effects on β-cell function in healthy individuals [23]. Although secretion of insulin was not directly assessed in this study, the observations that proinsulin (data not shown) and c-peptide responses mirrored the circulating insulin responses, along with the comparable β-cell function index across interventions, suggest that the MCT-induced ketosis did not potentiate the breakfast-stimulated insulin secretion. However, augmented PYY and GIP responses were noted in both MCT and MCT + WPI, although the pattern of the temporal responses for GIP (being higher in late breakfast postprandial period and early lunch postprandial period) suggests that it may not be directly implicated with the early attenuation of PPG observed after breakfast. Furthermore, unlike GLP1, the insulinotropic action of GIP is impaired in T2D [24]. In contrast, there is increasing evidence that PYY plays a key role in blood glucose regulation and pancreatic function in addition to its classical effect on appetite regulation [25]. Importantly, PYY appears to modulate glucose-stimulated insulin secretion chronically [26] but not acutely [27]. Hence, the extent to which chronic use of MCT preloads can modify PPG through tonic regulation of PYY function in patients with T2D remains to be investigated.

Another potential mechanism underpinning the glucose-lowering effect of MCT is the slowing of gastric emptying [28]. Although gastric emptying was not evaluated in this study, it is plausible that the tendency for higher GLP1 responses in MCT and MCT + WPI treatments observed when considering only the 18 completers might play a part in reducing gastric emptying [29]. However, not all studies support the involvement of endogenous GLP1 in gastric emptying in individuals with T2D [30]. PYY has also been shown to decrease gastric emptying [31], whereas GIP does not seem to have such an effect in healthy humans [32]. However, there is a gap in research data regarding individuals with T2D.

The beneficial impacts of whey protein as a preload in individuals with T2D [3,4,33] have been linked to diverse mechanisms, including slowing of gastric emptying and/or delayed glucose absorption in the small intestine [34,35], improved postprandial insulinaemia stemming from enhanced β-cell function and/or reduced insulin clearance [36], as well as enhanced secretion of incretin peptides such as GLP1, GIP, and PYY [[37], [38], [39], [40]]. Previous research indicated that 10 g of whey protein was the lowest preload dose administered 30 min before a meal to show a significant effect on PPG [41]. This dose was also used in this study, where 10 g of WPI consumed 30 min before lunch and dinner complemented the impact of MCT ingested before breakfast leading to a sustained reduction in PPG after breakfast and lunch in the MCT + WPI intervention, although it had no impact on diurnal or 24-h glycaemia.

Similar to this study, whey protein consumption has been shown to induce secretion of both insulin [42] and glucagon [43], with both responses correlating positively with elevated plasma amino acids concentrations. Hence, in this study, the whey protein contribution to the reduction in PPG can be attributed, at least partially, to the early but transient increase in insulin levels observed after the lunch preload. This insulin response coincided with elevated circulating levels of selected amino acids (including BCAA, arginine, alanine, and glutamate), recognized for their potent effect on stimulating secretion of insulin [44], and incretin peptides (GIP and PYY, and to a lesser extent GLP1). However, PYY does not seem to exert an acute impact on glucose-stimulated insulin secretion [27], the insulinotropic action of GIP is impaired in T2D [24], and GLP1 responses tended to be higher in the MCT + WPI treatment when only the 18 completers were considered in this study. Therefore, it is plausible that the reduction in PPG induced by whey protein is primarily mediated through the insulinotropic effect linked to the concurrent increase in plasma amino acids concentrations.

In this study, all 3 interventions were highly standardized. Specifically, all participants reported similar amounts and intensities of physical activities, their dietary energy intake and macronutrient composition were consistent, and they exhibited similar 24-h glycaemia for both run-in days preceding each intervention. However, some limitations need to be acknowledged. First, the measurements were conducted over 24 h, limiting the assessment of preloads’ effects on long-term indices of glycaemic control (such as HbA1c), and free-living energy intake. Second, the preloads (water) in CONTROL were not isoenergetic to the MCT and WPI preloads used in the other 2 interventions. Although it is challenging to fully differentiate the specific effects of MCT and WPI preloads, a potential impact of increased preload-associated energy provision alone on PPG is likely to be limited. However, it could also be argued that this aspect of the experimental design might enhance the generalizability of the current findings, as it reflects the typical pattern of preload ingestion under real-world conditions. Additional factors enhancing the generalizability of the findings include the physiologically relevant amounts of MCT and whey protein preloads ingested across various meals by both male and female participants.

In summary, in individuals with T2D, sequential oral intake of MCT preload at breakfast and WPI preloads at lunch and dinner for 1 day did not affect diurnal or 24-h glycaemia but resulted in reductions in PPG and 24 h glycaemic variability, both important contributors to chronic glycaemic control and development of microvascular complications. Future studies should investigate the long-term effects of daily MCT and WPI preload consumption on markers of glycaemic control (such as HbA1c) and appetite to provide further insights for the management of glycaemia and body weight in individuals with T2D.

## Author contributions

The authors’ responsibilities were as follows – LGK, KT: designed the studies; the project grant was awarded to KT; PP, JM, AA, SC, MK, MM, MJ, SB, LGK, KT: conducted research; KT, PP, MK, MM, MJ, OEJ: analyzed data and performed statistical analysis; KT, PP: wrote the original manuscript; and JM, AA, MK, SC, MM, MJ, SB, LGK, OEJ, BC: reviewed and approved the manuscript before submission.

## Funding

This research was supported by Nestlé Health Science via a project grant awarded to KT.

## Data availability

Data described in the manuscript, code book, and analytic code will be made available upon request.

## Conflict of interest

OEJ, BC, and LGK are or were employees of Société des Produits Nestlé, Lausanne, Switzerland, during the project. LGK is on the scientific advisory boards of Vital Proteins and NUUN, has participated on advisory boards of Liquid I.V., and has received personal fees from RNWY and Nestlé Health Science, and is a board member of Siftlink. The other authors report no potential conflicts of interest.
